# Effects of Walking Practice in Menopausal Women Monitored Using a Mobile Application

**DOI:** 10.1177/26924366251394595

**Published:** 2025-11-11

**Authors:** Yun-Su Kim

**Affiliations:** Department of Nursing, Honam University, Gwangju, Republic of Korea.

**Keywords:** walking program, mobile app, menopausal women, sleep, depression, self-efficacy

## Abstract

**Background:**

Physical activity has been shown to improve the health of middle-aged women and prevent menopause-associated disorders. Considering advances in technology, investigating the effects of exercises, especially walking, on the health of menopausal women using a mobile application is crucial. Therefore, this study aimed to implement a 12-week walking program for menopausal women using a mobile application (WalkON) and to investigate changes in health conditions, lifestyle habits, social support and mental health, health-promoting behaviors, health beliefs, and self-efficacy.

**Methods:**

This observational study, utilizing a nonequivalent control group and pre- to post-test design, was conducted between September 9, 2024, and November 11, 2024, with the intervention group consisting of 46 participants and the control group consisting of 45 participants. SPSS software was used to analyze the data.

**Results:**

The findings revealed that, compared with those in the control group, sleep quality scores in the intervention group decreased significantly following the walking intervention, indicating that sleep quality improved in the intervention group (*p* < 0.05). In addition, changes in depression levels were also significant between the two groups (*p* < 0.001). Among the health-promoting behavior subfactors, there was a significant difference between the two groups in the health responsibility domain (*p* < 0.01). There was also a significant difference in the nutrition domain between the intervention and control groups (*p* < 0.05). Similarly, a significant difference between the two groups was found in the self-efficacy domain (*p* < 0.05).

**Discussion:**

WalkONs have potential positive effects on health-promoting behaviors and mental health improvement, and could serve as an effective strategy to improve physical and mental health in women experiencing menopausal symptoms.

## Introduction

Recent advances in science and health care technology, along with improved living standards, have led to an increase in life expectancy in modern society. On December 23, 2024, South Korea officially qualified as a “super-aged society,” defined as a nation in which >20% of the total population is aged 65 years or older. It is estimated that women aged 65 years or older may constitute 22.73% of the total female population in May 2025, which might reach 39.8% by 2050.^1^

The health of middle-aged women, who often play pivotal domestic roles, is a crucial issue—not only from a personal perspective but also because it impacts the health of families, regions, and the nation as a whole.^2^ Key focus areas for middle age include addressing the rapid rate of aging, managing physical and psychological changes, adapting to evolving marital relationships, adjusting to changes in one’s roles in family function and structure, and preparing for active old age.^3^ Menopause, a life stage experienced by middle-aged women, can also act as a physiological, psychological, or social crisis in their lives.^4^ During this period, women experience various changes, including menopausal symptoms such as fatigue, sleep disturbances, hot flashes, and headaches, as well as changes in sexual emotions, attitudes, and relationships.^5^ These symptoms negatively affect the lives of menopausal women and may present psychologically as depression, anxiety, or restlessness.^6^

Currently, the mean age of menopause among South Korean women is 49.9 years,^7^ with menopausal symptoms beginning 4–6 years before menopause and persisting for several years.^8^ Neglecting these symptoms results in an increased prevalence of postmenopausal secondary diseases, including metabolic syndrome and dyslipidemia.^9^ Middle age, a period that is predictive of life in old age, is a critical time when regular physical activity becomes essential for healthy aging.^10^ As of 2024, the rate of physical activity among South Korean women (46.0%) is lower than that among men (50.7%).^11^ This percentage is less than half of the World Health Organization (WHO) recommended criterion—moderate activity for at least 150 min/week—and has shown a decreasing or static trend.^12^

Increased aerobic physical activity among middle-aged women is not only associated with improved physical health^13^ but also positively affects social and mental health, including anxiety and depression.^14^ Engaging in merely 50 min of physical activity, twice a week for 10–12 weeks, can effectively alter body weight and fat in menopausal women, thereby improving sleep quality and alleviating incontinence and depression.^15,16^ A 30-year study on middle-aged women found that consistent walking reduced the incidence of cardiovascular disease by 33%.^17^ Compared with the “no walking exercise” group, the “regular walking exercise” group exhibited significant differences in mental stress, pain, discomfort, sleep duration, and subjective health status.^18^ Additionally, middle-aged women who performed regular physical activity showed significant differences in self-efficacy and depressive symptoms.^19^ Therefore, increasing physical activity is considered one of the most important contributors to improving the health of middle-aged women, and sustained, regular physical activity can potentially prevent future diseases.

Health-promoting behaviors aim to achieve an optimal state of well-being and satisfaction, and to maintain and improve personal needs and self-realization. These behaviors are influenced by various factors, including physical, biological, and social environments, lifestyle patterns, and health management systems. Health beliefs—personal and subjective beliefs—are key factors in predicting and explaining health-related behaviors. They involve a behavioral evaluation of predicted future outcomes based on the interaction between perceived threat—the expectation that one will develop a disease—and healthy behavior.^20^ Self-efficacy is defined as the ability to perform healthy behaviors, including the level of effort required to achieve one’s goals, the power and autonomy to avoid risky behaviors, and the ability to strategically overcome the causes of disability.^21^ Therefore, self-efficacy and health beliefs are important variables that can improve health-promoting activities.

Walking is the simplest aerobic exercise to perform daily, and it greatly improves basic fitness while reducing body fat. As a representative low-impact aerobic exercise, walking is recommended to improve health in both men and women, as it places less stress on skeletal muscles and joints than running and minimizes potential injury while still providing the benefits of exercise.^22^ In addition to preventing chronic diseases such as hypertension and diabetes, exercise is also potentially effective in alleviating psychiatric conditions such as insomnia and depression.^23^

The mobile WalkON application is an established walking-based health care platform designed to increase users’ physical activity levels, particularly through walking. The app is publicly available and free for anyone to use. Additionally, users can create a community of dedicated members and operate paid programs (https://www.swallaby.com, 27 Teheran-ro 2-gil, Gangnam-gu, Seoul, Republic of Korea). Using a smartphone or wearable device (watch, band, etc.), users’ daily walking logs are automatically stored, and health-related data such as the daily number of steps, distance traveled, calories burned, walking time, and sleep can be viewed at a glance. Promoting physical activity can help maintain overall health. If users input their sex, age, height, and weight into their profile, they will be informed of their personally recommended activity level. By participating in walking challenges by region, organization, or company and achieving their target number of steps, users can receive various forms of compensation, such as coupons. Users can also create walking communities together with family, friends, workplaces, or local residents and compare step counts or participate in small group activities. Within these communities, motivation can be enhanced through walking acknowledgments, rankings, and badges. Based on users’ locations, the application recommends nearby walking routes, courses, and sites to help them discover new walking paths. The application also provides videos and informative content to support health education and management. Currently, official walking communities and challenges are managed by local governments, companies, and schools, and these applications are used to promote health improvement and prevent chronic diseases among local residents. Many users have provided positive feedback, reporting increased interest and motivation through goal setting, a sense of achievement, compensation, and community activities compared with those performing walking exercises alone. Since the COVID-19 pandemic, noncontact environments are expected to become the basis for new standards and values. Hence, forming physical activity habits through a noncontact health management platform is expected to be helpful in building health management systems (https://www.swallaby.com). Therefore, it is necessary to verify whether menopausal women can maintain physical vitality and healthy mental well-being through walking activities via mobile apps in their daily lives, participate in challenges through small group activities, and utilize various health information.

The objective of this study was to implement a 12-week walking program for menopausal women using a mobile application (WalkON) and to investigate the changes in health conditions, lifestyle habits, social support and mental health, health-promoting behaviors, health beliefs, and self-efficacy.

## Methods

### Study design

**FIG. 1.**
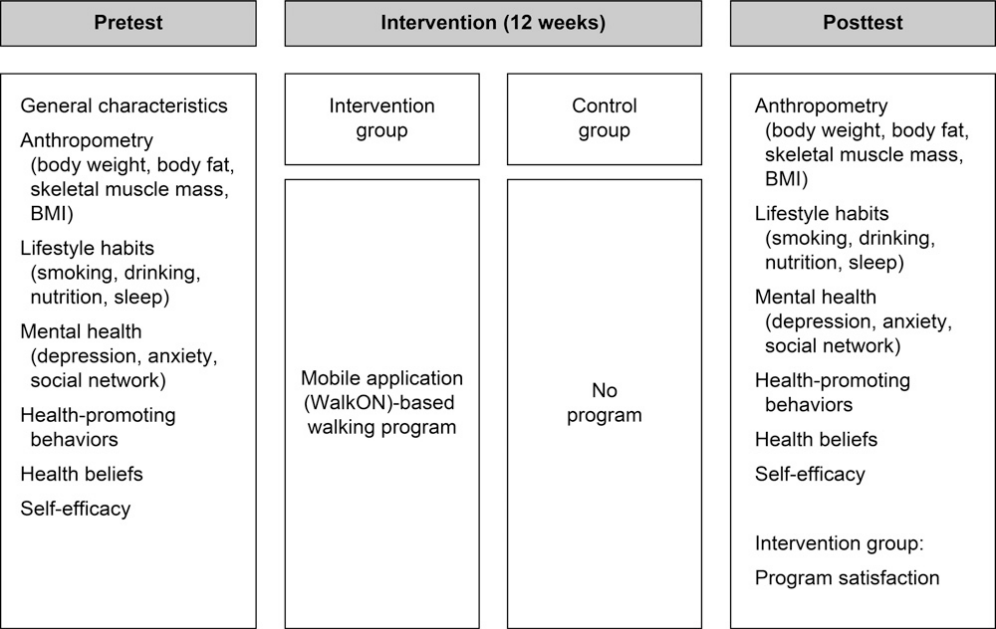
Schematic representation of the study design.

In terms of methodology, this was a quasi-experimental study involving a nonequivalent control group and a pre- to post-test design. The independent variable was 12 weeks of walking activity monitored using the mobile application WalkON, while the dependent variables were health-related habits (sleep, nutrition, smoking, and drinking); mental health and social environment status (depression, anxiety, and social networks); health-promoting behaviors; health beliefs; self-efficacy; and body measurement results. WalkON is a free health management platform that encourages walking activities based on personal physical activity data analyzed through smartphones, helping users develop and maintain healthy lifestyle habits. The intervention group underwent anthropometric measurements, including body fat, body weight, skeletal muscle mass, and visceral fat levels, and completed a questionnaire before participating in the 12-week walking program. Participants were encouraged to walk for at least 30 min for at least 5 days per week. During the program, physical activity was promoted by posting educational materials for health improvement on the application’s message board and hosting a total of three 4-week challenges to encourage walking. After completing the program, participants completed the same questionnaire and underwent anthropometric measurements as in the pretest, and their satisfaction with the program was also assessed. The control group completed the same pretest questionnaire and anthropometric measurements as the study participants and repeated the same questionnaire and assessments 12 weeks later (Fig. 1).

### Participants

The study participants were menopausal women aged 45–60 years. Participants were recruited through social media bulletin boards commonly used by the target age group, as well as through referrals from the researchers’ acquaintances. The inclusion criteria were middle-aged women aged 45–60 who owned a smartphone, were able to participate in a 12-week walking program using a mobile application, and understood the purpose of the study and agreed to participate. Individuals with special health problems that prevented them from walking for 12 weeks were excluded. Participants were divided into a group that participated in the walking program using a mobile application (*n* = 46) and a group that did not (*n* = 45). The study duration was 12 weeks, from September 9, 2024, to November 30, 2024. Using G*Power and assuming a moderate effect size of 0.6, a significance level of 95%, and explanatory power of 80%, the minimum required sample size was calculated to be 40 participants per group. At the start of the study, there were 46 and 45 participants in the intervention and control groups, respectively, and all participants completed the study without dropping out.

### Research instruments

#### Assessment of lifestyle habits

Smoking was assessed using a total of nine questions, including past smoking quantity and duration, current smoking quantity and duration, abstinence intention, self-confidence in abstinence, and nicotine dependence.^24^ Drinking was assessed using 10 questions from an instrument developed by the WHO,^25^ covering hazardous drinking and symptoms of alcohol dependence. Exercise was assessed by asking about physical activity performed during the past 7 days.^26^

Nutrition was assessed using the Healthy Diet Index, which reflects the quality of meals for South Koreans.^27^ Sleep quality was assessed over 1 month using the PSQI, validated for Koreans. The PSQI consists of 19 questions related to sleep quality, duration, efficiency, disturbances, use of sleeping medication, and impaired daytime functioning. The total score ranges from 0 to 21 points,^28^ with higher scores indicating a worse quality of sleep. In terms of reliability, Cronbach’s α was 0.83 at the time of development and 0.76 in this study.

#### Mental health and social support assessment

The Patient Health Questionnaire-9 (PHQ-9) was used to measure depression, adapted and standardized by Donnelly^29^ from the PHQ-9 originally developed by Kroenke et al.^30^ The PHQ-9 measures the level of depression over the past 2 weeks and is a simple method for screening and assessing depression and its severity. Cronbach’s α was 0.86 in this study.

The Beck Anxiety Inventory was used to assess anxiety, adapted by Kwon^31^ from the system that was originally developed by Beck et al. Through this questionnaire, participants indicated their level of anxiety over the past week via 21 items related to the cognitive, emotional, and physical aspects of anxiety. Cronbach’s α was 0.91 in a study by Kim and Yook^32^ and 0.92 in this study.

Social networks were assessed using the Lubben Social Network Scale.^33^ This system measures bonds and relationships involving the continual and mutual exchange of service and assistance through contact and socialization with family, friends, and neighbors. Higher scores indicate greater social support. In terms of reliability, Cronbach’s α was 0.74 in this study.

#### Health-promoting behaviors

Health-promoting behaviors were measured using a questionnaire adapted by Eom^34^ from the Health-Promoting Lifestyle Profile II developed by Walker et al.^35^ This questionnaire consists of 52 items measured on a 4-point scale with higher scores indicating higher levels of health-promoting behaviors. In terms of reliability, Cronbach’s α was 0.94 at the time of development by Walker et al.^35^ and 0.94 in this study.

#### Health beliefs

Health beliefs were assessed using a questionnaire originally developed by Walker et al.^35^ and adapted for the Korean population by Lee.^36^ This questionnaire evaluates 22 parameters, which include perceived sensitivity, severity, benefits, and barriers. In terms of reliability, Cronbach’s α was 0.75 in this study.

#### Self-efficacy

Self-efficacy was measured using a 17-item questionnaire developed by Sherer et al.^37^ and adapted by Kim et al.^38^ Cronbach’s α was 0.90 in the study by Kim et al.^38^ and 0.97 in this study.

### Data collection

Prior to the collection of the relevant data, the study was approved by the institutional review board of Honam University (Honam University 1041223-202406-HR-10). The study objectives and program content were explained to the participants, and written informed consent was obtained from all participants.

### Data analyses

Questionnaire survey results and anthropometric measurements were statistically analyzed using SPSS version 25. Statistical analyses included basic statistics to ascertain the participants’ characteristics, as well as chi-square tests, paired *t*-tests, and unpaired *t*-tests.

## Results

### General participant characteristics

Table 1 presents the general characteristics of the intervention and control groups; there were no significant differences between the two groups. In the intervention group, 24 women (52.2%) confirmed that they were currently enduring a disease. The most commonly reported conditions were hypertension (*n* = 4, 8.7%), hyperlipidemia (*n* = 7, 15.2%), diabetes (*n* = 3, 6.5%), hypothyroidism (*n* = 3, 6.5%), and osteoporosis (*n* = 2, 4.3%). Other reported conditions included arthritis, hyperthyroidism, cervical disk herniation, and lymphedema. In addition, 22 women (47.8%) reported no current diseases.

**Table 1.** General and Health-Related Characteristics of Study Participants

Variables	Exp (*N* = 46)	Cont (*N* = 45)	*t* or χ^2^	*p*
	Mean ± standard deviation of *n* (%)			
Age	52.54 ± 0.63	51.22 ± 0.55	0.15	0.880
Age at menopause	50.73 ± 0.72	50.58 ± 0.62	0.11	0.170
Experience of childbirth				
Yes	44 (95.7)	42 (93.3)	0.24	0.627
No	2 (4.3)	3 (6.7)		
Number of children				
None	2 (4.3)	3 (6.7)	2.04	0.564
1	8 (17.4)	5 (11.1)		
2	30 (65.2)	27 (60.0)		
3 or more	6 (13.0)	10 (22.2)		
Current menopausal status				
Premenopause	14 (30.4)	19 (42.2)	1.91	0.384
Perimenopause	6 (13.0)	7 (15.6)		
Postmenopause	26 (56.5)	19 (42.2)		
Menopausal symptoms				
Pain	2 (6.7)	2 (4.4)	4.05	0.866^a^
Sleep impairment	3 (10.0)	1 (2.2)		
Forgetfulness, memory decline	5 (16.7)	1 (2.2)		
Increased abdominal circumference and weight gain	9 (30.0)	6 (13.3)		
Cold sweats	3 (10.0)	2 (4.4)		
Blushing	0 (0.0)	1 (2.2)		
Other	2 (4.4)	0 (0.0)		
No symptoms	7 (23.3)	5 (11.1)		
Marital status				
Unmarried	0 (0.0)	3 (6.7)	3.44	0.179
Married	43 (93.5)	38 (84.4)		
Other	3 (6.5)	4 (8.9)		
Educational attainment				
Middle school graduation or below	0 (0.0)	1 (2.2)	4.60	0.203
High school graduation	4 (8.7)	9 (20.0)		
University graduation	24 (52.2)	24 (53.3)		
Graduate school graduation or above	18 (39.1)	11 (24.4)		
Area of residence				
Metropolitan city/Special city	41 (89.1)	38 (84.4)	0.50	0.758
Medium-sized city	4 (8.7)	6 (13.3)		
County	1 (2.2)	1 (2.2)		
Occupation				
Full-time homemaker	9 (19.6)	10 (22.2)	2.71	0.745
Sales/service	3 (6.5)	6 (13.3)		
Professional role	14 (30.4)	14 (31.1)		
Administrative role	1 (2.2)	1 (2.2)		
Clerical role	9 (19.6)	9 (20.0)		
Other	10 (21.7)	5 (11.1)		
Height (cm)	162.31 ± 5.34	159.77 ± 5.34	0.08	0.200
Weight (kg)	58.06 ± 9.66	57.87 ± 6.76	0.08	0.200
Body mass index (kg/m^2^)	22.61 ± 3.79	22.74 ± 2.67	0.09	0.200
Body fat percentage (%)	28.64 ± 7.05	30.54 ± 5.36	0.09	0.200
Skeletal muscle mass (kg)	22.23 ± 2.61	20.29 ± 4.76	0.09	0.200
Mobile application use				
Total participation time (days)	80.67			
Total step count (steps)	452,784			
Mean step count (steps/day)	5613			

^a^ Fisher’s exact test.

Cont, control group; Exp, experimental group.

In the control group, 20 women (44.4%) reported that they were currently experiencing a disease, with the most commonly reported diseases being hypertension (*n* = 3, 6.7%), hyperlipidemia (*n* = 7, 15.6%), diabetes (*n* = 3, 6.7%), and arthritis (*n* = 3, 6.7%). Other reported conditions included scoliosis, uterine myoma, hypothyroidism, fatty liver disease, esophagitis, and sudden sensorineural hearing loss. In the control group, 25 (55.6%) women reported no current disease (Table 1).

In addition, there were no significant differences between the two groups in mean weight, body mass index (BMI), body fat percentage, or skeletal muscle mass (Table 1).

One woman each in the intervention (2.0%) and control (1.9%) groups was a current smoker, with a smoking frequency of <10 cigarettes per day and an intention to quit smoking within 6 months. In terms of drinking habits, three participants each in the intervention (6.0%) and control (5.8%) groups reported consuming alcohol two to three times per week. For the question “In the last year, have you ever started drinking and been unable to stop yourself?,” three participants in both the intervention (6.0%) and control (5.8%) groups reported this occurring approximately once a week.

The number of days of participation in the program, total number of steps per participant, and average number of steps per day using the mobile app (WalkON) for the intervention group are shown in Table 1. Power walking refers to walking at a pace of >80 steps/min (https://www.swallaby.com).

### Testing the homogeneity of the dependent variables

The homogeneity of the dependent variables was confirmed: the intervention and control groups showed no significant differences in lifestyle habits, social networks, mental health, or health-promoting behaviors. The Mann–Whitney *U* test was used to analyze depression and anxiety because these variables did not satisfy the assumption of normality; however, no significant differences were identified (*p >* 0.05), thus confirming homogeneity between the groups (Table 2).

**Table 2.** Homogeneity Testing of the Main Variables Between Groups

Variables	Exp (*N* = 46)	Cont (*N* = 45)	*t*/*u*	*p*
	Mean ± SD			
Nutrition	20.91 ± 4.72	22.40 ± 5.09	0.10	0.200
Sleep	5.74 ± 1.40	5.60 ± 2.53	0.96	0.076
Depression	2.72 ± 2.58	2.62 ± 3.40	0.20	0.284^a^
Anxiety	5.11 ± 6.39	5.98 ± 6.84	0.21	0.168^a^
Social network	23.85 ± 6.31	23.73 ± 6.44	0.09	0.200
Health-promoting behaviors	2.41 ± 0.45	2.41 ± 0.42	0.11	0.200
Health responsibility subdomain	2.18 ± 0.46	2.11 ± 0.50	0.09	0.200
Physical activity subdomain	2.31 ± 0.83	2.16 ± 0.71	0.09	0.200
Nutrition subdomain	2.32 ± 0.52	2.48 ± 0.56	0.10	0.200
Interpersonal relationships subdomain	2.64 ± 0.53	2.62 ± 0.45	0.10	0.200
Spiritual growth subdomain	2.69 ± 0.61	2.69 ± 0.61	0.09	0.200
Stress management subdomain	2.29 ± 0.57	2.38 ± 0.53	0.08	0.200
Health beliefs	3.40 ± 0.42	3.45 ± 0.35	0.13	0.059
Self-efficacy	3.76 ± 0.63	3.78 ± 0.63	0.08	0.200

^a^ Mann–Whitney *U* test.

Exp, experimental group; Cont, control group.

### Lifestyle habit-related effects of WalkON-based walking program for menopausal women

In terms of changes in body weight, body fat, BMI, or skeletal muscle mass, there were no notable differences between the two groups after the program intervention (Table 3).

**Table 3.** Lifestyle Habit-Related Effects of the Walking Program in the Intervention and Control Groups

Variables	Pretest	Post-test	Unpaired *t*	*p*	Difference	Paired *t*	*p*
	Mean ± SD				Mean ± SD		
Weight (kg)							
Exp	58.06 ± 9.66	58.04 ± 9.39	0.11	0914	−0.019 ± 1.32	−1.29	0.205
Cont	57.87 ± 0.76	58.24 ± 6.86	−0.11	0.910	0.13 ± 0.54		
Body fat percentage (%)							
Exp	28.64 ± 7.05	29.12 ± 6.66	−1.44	0.152	0.25 ± 2.48	1.11	0.273
Cont	30.54 ± 5.36	30.45 ± 4.81	−1.08	0.282	−0.09 ± 1.96		
Skeletal muscle mass (kg)							
Exp	22.23 ± 2.61	22.26 ± 2.80	2.42	0.018	0.02 ± 1.22	0.51	0.611
Cont	20.29 ± 4.76	20.19 ± 4.81	2.52	0.014	−0.10 ± 1.31		
BMI (kg/m^2^)							
Exp	22.61 ± 3.79	22.20 ± 4.98	−0.19	0.844	−0.41 ± 3.26	−0.95	0.345
Cont	22.74 ± 2.67	22.81 ± 2.62	−0.72	0.473	0.06 ± 0.65		
Nutrition							
Exp	20.91 ± 4.72	24.41 ± 3.37	−1.44	0.152	3.51 ± 7.14	−1.49	0.145
Cont	22.40 ± 5.09	23.73 ± 3.06	1.01	0.318	1.33 ± 7.43		
Sleep							
Exp	5.74 ± 1.40	4.37 ± 0.97	0.33	0.746	1.31 ± 0.90	2.45	0.018^*^
Cont	5.60 ± 2.53	4.89 ± 1.89	−1.64	0.103	0.71 ± 1.35		
Social network							
Exp	23.85 ± 6.31	24.30 ± 6.19	0.09	0.932	0.42 ± 4.36	−0.24	0.811
Cont	23.73 ± 6.44	24.44 ± 6.80	−0.10	0.918	0.71 ± 5.82		

^*^ *p* < 0.05.

^**^ *p* < 0.01.

^***^ *p* < 0.001.

BMI, body mass index; Cont, control group; Exp, experimental group.

For nutritional status, the mean total score (out of 55 points) increased from 20.91 points preintervention to 24.41 points postintervention. The control group also showed an increase from 22.40 points to 23.73 points; however, the difference between the two groups was not statistically significant (*t* = −1.49, *p* = 0.145).

In the intervention group, sleep quality scores decreased from 5.74 points preintervention to 4.37 points postintervention, indicating an improvement in sleep quality. In the control group, sleep quality scores decreased from 5.60 to 4.89 points. The change in sleep quality differed significantly between the two groups (*t* = 2.45, *p* = 0.018).

Social network scores, representing social support, increased from 23.85 points preintervention to 24.30 points postintervention in the intervention group, and the control group also showed an increase from 23.73 to 24.44 points. The difference between the groups was not significant (*t* = −0.24, *p* = 0.811; Table 3).

### Mental health-related effects of the mobile application-based walking program for menopausal women

In the intervention group, depression scores decreased from 2.72 points preintervention to 1.67 points postintervention, whereas the control group showed an increase from 2.62 to 2.73 points. Thus, the intervention program led to a significant increase in depression scores (*u* = −3.49, *p* < 0.001).

Anxiety scores also decreased in the intervention group from 5.11 points preintervention to 4.65 points postintervention, while those in the control group decreased from 5.98 to 5.51 points, showing no significant difference in the changes in anxiety scores between the two groups (*u* = 0.00, *p =* 1.000; Table 4).

**Table 4.** Mental Health-Related Effects of the Walking Program in the Intervention and Control Groups

Variables	Pretest	Post-test	*u*	*p*	Difference	*u*	*p*
	Mean ± SD				Mean ± SD		
Depression							
Exp	2.72 ± 2.58	1.67 ± 1.91	963.50	0.564	−1.04 ± 1.20	−3.49	0.000^***^
Cont	2.62 ± 3.40	2.73 ± 4.09	928.00	0.377	0.11 ± 1.61		
Anxiety							
Exp	5.11 ± 6.39	4.65 ± 5.29	914.00	0.334	−0.46 ± 3.95	0.00	1.000
Cont	5.98 ± 6.84	5.51 ± 7.53	1004.50	0.807	−0.46 ± 3.73		

^*^ *p* < 0.05.

^**^ *p* < 0.01.

^***^ *p* < 0.001.

Cont, control group; Exp, experimental group.

### Health-promoting behavior-related effects of the mobile application-based walking program for menopausal women

The overall level of health-promoting behaviors increased in both groups, with an increase in the intervention group from 2.41 points preintervention to 2.51 points postintervention, while the control group showed an increase from 2.41 points to 2.45 points. There was no significant difference between the two groups (*t* = 1.22, *p* = 0.229; Table 5).

**Table 5.** Health-Promoting Behavior-Related Effects of the Walking Program in the Intervention and Control Groups

Variables	Pretest	Post-test	Unpaired *t*	*p*	Difference	Paired *t*	*p*
	Mean ± SD				Mean ± SD		
Health-promoting behaviors							
Exp	2.41 ± 0.45	2.51 ± 0.46	0.01	0.992	0.09 ± 0.19	1.22	0.229
Cont	2.41 ± 0.42	2.45 ± 0.45	0.64	0.552	0.03 ± 0.27		
Health responsibility							
Exp	2.18 ± 0.46	2.46 ± 0.44	0.74	0.461	0.27 ± 0.19	2.92	0.005^**^
Cont	2.11 ± 0.50	2.21 ± 0.48	2.58	0.012	0.09 ± 0.35		
Physical activity							
Exp	2.31 ± 0.83	2.33 ± 0.74	0.19	0.356	0.02 ± 0.43	−1.11	0.274
Cont	2.16 ± 0.71	2.28 ± 0.75	0.34	0.734	0.12 ± 0.44		
Nutrition							
Exp	2.32 ± 0.52	2.48 ± 0.52	−1.44	0.152	0.14 ± 0.39	2.35	0.023^*^
Cont	2.48 ± 0.56	2.44 ± 0.55	0.32	0.750	−0.04 ± 0.34		
Interpersonal relationships							
Exp	2.64 ± 0.53	2.71 ± 0.47	0.19	0.847	0.06 ± 0.27	0.74	0.464
Cont	2.62 ± 0.45	2.64 ± 0.48	0.70	0.485	0.01 ± 0.35		
Spiritual growth							
Exp	2.69 ± 0.61	2.74 ± 0.61	−0.04	0.997	0.04 ± 0.31	0.79	0.431
Cont	2.69 ± 0.61	2.67 ± 0.53	0.58	0.565	−0.01 ± 0.45		
Stress management							
Exp	2.29 ± 0.57	2.44 ± 0.54	−0.75	0.457	0.13 ± 0.28	1.64	0.108
Cont	2.38 ± 0.53	2.40 ± 0.55	0.28	0.783	0.02 ± 0.36		
Health beliefs							
Exp	3.40 ± 0.42	3.37 ± 0.34	−0.64	0.526	−0.62 ± 1.06	0.13	0.899
Cont	3.45 ± 0.35	3.43 ± 0.33	−0.75	0.458	0.77 ± 0.87		
Self-efficacy							
Exp	3.76 ± 0.63	4.07 ± 0.62	−0.13	0.897	0.24 ± 0.27	2.08	0.044^*^
Cont	3.78 ± 0.63	3.83 ± 0.74	1.19	0.236	0.05 ± 0.57		

^*^ *p* < 0.05.

^**^ *p* < 0.01.

^***^ *p* < 0.001.

Cont, control group; Exp, experimental group.

Among the subdomains of health-promoting behaviors, mean scores in the health responsibility domain increased from 2.18 points preintervention to 2.46 points postintervention in the intervention group, and from 2.11 to 2.21 points in the control group. The intervention induced a significant increase in health-promoting behaviors between the two groups (*t* = 2.92, *p* < 0.01).

In the physical activity domain, mean scores in the intervention group increased from 2.31 points preintervention to 2.33 points postintervention, while the control group showed an increase from 2.16 to 2.28 points. The difference between the groups was not significant (*t* = −1.11, *p* = 0.274).

In the nutrition domain, mean scores in the intervention group increased from 2.32 points preintervention to 2.48 points postintervention, whereas the control group showed a decrease from 2.48 to 2.44 points (*t* = 0.32, *p* = 0.750). The difference between the two groups was statistically significant (*t* = 2.35, *p* < 0.05).

In the interpersonal relationships domain, mean scores in the intervention group increased from 2.64 points preintervention to 2.71 points postintervention, while the control group increased from 2.62 to 2.64 points; however, the difference between the two groups was not significant (*t* = 0.74, *p* = 0.464).

In the spiritual growth domain, mean scores in the intervention group increased from 2.69 points preintervention to 2.74 points postintervention, while the control group decreased from 2.69 to 2.67 points; however, the difference between the two groups was not significant (*t* = 0.79, *p* = 0.431).

In the stress management domain, mean scores in the intervention group increased from 2.29 points preintervention to 2.44 points postintervention, while the control group increased from 2.38 to 2.40 points. Statistically, the difference between the two groups was not significant (*t* = 1.64, *p* = 0.108; Table 5).

For the level of health beliefs, mean scores in the intervention group decreased from 3.40 points preintervention to 3.37 points postintervention, while the control group decreased from 3.45 to 3.43 points. There was no significant difference between the two groups (*t* = 0.13, *p* = 0.899; Table 5).

The mean self-efficacy scores in the intervention group increased from 3.76 points preintervention to 4.07 points postintervention, while the control group showed an increase from 3.78 to 3.83 points. Thus, after the intervention, the change in self-efficacy scores was significantly different between the two groups (*t* = 2.08, *p* < 0.05; Table 5).

### Program satisfaction survey

In a satisfaction survey administered to the intervention group, for the statement, “participating in the program was a valuable experience,” the mean score was 4.30 ± 0.69 out of 5 points. For the statement “I was able to perform behaviors for health improvement through the program,” the mean score was 4.26 ± 0.74 points. For the statement “While participating in the program, I felt a sense of achievement,” the mean score was 4.04 ± 0.73 points. For the statement “walking time while using the mobile app was good,” the mean score was 4.20 ± 0.72 points. For the statement “I would actively recommend walking practice using the mobile application,” the mean score was 4.26 ± 0.74 points.

## Discussion

In this study, a mobile application-based (WalkON) 12-week walking program was implemented for menopausal women to explore specific strategies for promoting healthy behaviors. A daily walking count of 7000 steps is known to lower the risk of premature mortality by 50–70% in middle-aged women compared with women walking fewer steps,^39^ and a step count interval of 6000–8000 steps/day is considered most effective in preventing chronic diseases.^40^ A meta-analysis of data from 227,000 participants across 17 studies in six countries (the United States, UK, Australia, Japan, Norway, and Spain) revealed that the risk of death from cardiovascular disease begins to decrease at ≥2337 steps/day, and at 4000 steps/day and above, the risk of death from all causes begins to decline. These health benefits tend to increase with higher step counts.^41^

In this study, the mean total step count per participant was 452,784 steps (power walk: 263,707 steps; normal walk: 189,077 steps), with a mean daily step count of 5613 steps. In terms of sleep quality scores, the control group decreased by 0.71 points and the intervention group by 1.31 points, with a statistically significant difference between the two groups. This improvement in sleep quality may be attributed to regular walking. In a meta-analysis of 21 studies reported that walking-based aerobic exercise significantly improved sleep quality, insomnia severity, and sleep efficiency in both middle- and older-aged participants. In particular, “brisk walking” is the most effective exercise for improving insomnia, with effects evident from 4 weeks after initiating exercise and further enhanced when exercise is maintained for 8–16 weeks.^42^

In a longitudinal study of 4399 people from nine European countries over 10 years, it was reported that people who consistently walked at least 2–3 times per week had a significantly lower insomnia risk and a higher likelihood of obtaining 6–9 h of normal sleep compared with those who did not engage in regular walking. However, these effects disappeared when the activity stopped, highlighting the importance of persistent practice.^43^

The walking intervention in this study significantly affected the depression scores of the two groups. A study of 6800 middle-aged participants from South Korea revealed that walking at least five times per week lowers the risk of depressive mood by 47% and the risk of suicidal ideation by 75% compared with participants who do not walk regularly.^44^ A meta-analysis of 33 studies revealed that walking at least 5000 steps a day was associated with a decrease in depressive symptoms, and walking at least 7000 steps/day decreased the risk of depression by 31%. An increase in 1000 steps/day was also linked with a 9% decrease in the relative risk of depression.^45^ In an 11-year observational study of a cohort of 33,908 healthy adults in Norway, exercise was estimated to decreased the risk of depression by 12%.^46^

Among the subdomains of health-promoting behaviors, the two groups differed significantly in terms of health responsibility and nutrition following the intervention. A study analyzing the effects of a walking exercise on participants’ health-promoting behaviors revealed that the psychological and educational satisfaction obtained from walking positively influenced the practice of health-promoting behaviors, particularly in the self-realization, stress management, exercise, and health responsibility subdomains.^47^

Self-efficacy scores also differed significantly between the two groups. A study that implemented a 12-week forest-walking exercise program for middle-aged women reported significant improvements in functional fitness and self-efficacy.^48^ In another study, a 10-week walking intervention for clerical workers significantly increased self-efficacy.^49^ Therefore, because walking is highly accessible in daily life and improves self-efficacy, it can be considered an effective health improvement strategy to help individuals consistently engage in physical activity. Menopausal women who participated in this study were provided with diverse information via the mobile application (WalkON), including guidance on walking exercises, exercise videos, educational materials related to diet and nutrition, and opportunities to participate in challenges to encourage walking. This approach is presumed to enhance self-efficacy not only by increasing the participants’ confidence in achieving their exercise goals but also by promoting behavioral changes through emotional engagement.

Although anxiety decreased in both the intervention and control groups, no significant difference was observed between the two groups. This outcome differs from a previous study in which positive effects on anxiety were observed in middle-aged women who walked at least three times per week compared with those who did not exercise.^15^

Based on the anthropometric measurements, BMI, body weight, body fat, and skeletal muscle mass did not differ notably between the two study groups. Considering previous studies in South Korea and abroad investigating body composition after walking exercise, walking was found to decrease cardiovascular risk factors in obese individuals.^50^ The administration of a 12-week walking exercise program to obese middle-aged women decreased their mean body weight by 8.25% and body fat percentage by 13.73%.^51^ Thus, the reductions were more pronounced at higher levels of obesity. This suggests that the present study participants who had average body weight, body fat, BMI, and skeletal muscle mass at baseline may not have exhibited significant changes in these parameters after 12 weeks of walking.

In a meta-analysis on increasing physical activity, behavioral interventions were more effective than cognitive interventions, and methods such as self-monitoring and setting behavioral targets were identified as effective strategies for implementing physical activity interventions.^52^ In the present study, participants could use the mobile application to visually monitor their daily step counts and calorie consumption. They could also form communities and share step counts, enabling autonomous competition. These features could be considered as valid strategies for promoting physical activity. The participants were continuously motivated to promote persistent physical activity, encouraging them to check their weekly step counts, update methods of alleviating menopausal symptoms, and other health information materials via the mobile application message board, and host walking challenges throughout the 12-week intervention.

This study has some limitations. First, as the participants were restricted to menopausal women in a specific region, the generalizability of the findings is limited. Moreover, as we were unable to block the intervention effects with certainty, caution is required when interpreting the results. Second, diet or personal management during the walking study could not be controlled. Third, invasive physiological indicators, such as fasting blood sugar, glycated hemoglobin, and cholesterol-related indicators, could not be assessed. Based on these study results, further studies are necessary to validate and verify the effects of the mobile application and to confirm the changes in physiological indices when using the app. In addition, further experimental studies should be conducted to evaluate the effects of walking programs using wearable devices to continue improving walking intervention strategies and increasing health-promoting behaviors among menopausal women.

## Conclusion

The 12-week mobile application (WalkON)-based walking intervention program was effective in enhancing sleep quality, reducing depression, improving health responsibility and nutrition subdomains of health-promoting behaviors, and increasing self-efficacy among menopausal women. Since the COVID-19 pandemic, noncontact environments have become the foundation for new values and strategies. In this context, this study is valuable because we verified the effects of an intervention designed to efficiently improve health-promoting behaviors using an IT-based mobile application. Over the 12-week period, encouraging participants to check their daily step counts and active times, provide health information on exercise, diet, and nutrition using a message board, offering strategies to improve menopausal symptoms, and organizing challenge activities for the participants proved to be effective strategic approaches. The results confirmed that the menopausal women in this study were able to use the mobile application (WalkON) to motivate themselves and voluntarily participate in daily walking. Based on the positive effects of the intervention, we anticipate that it can be widely used as a strategy for health improvement programs. We believe that empirical studies are required to further develop this approach into a more targeted and efficient program for promoting walking.

## Data Availability

The data that support the findings of this study are available upon reasonable request from the corresponding author. The data are not publicly available due to privacy or ethical restrictions.
